# TRIM37 exacerbates hepatic ischemia/reperfusion injury by facilitating IKKγ translocation

**DOI:** 10.1186/s10020-023-00653-2

**Published:** 2023-05-08

**Authors:** Hang Yang, Zuotian Huang, Yunhai Luo, Dengliang Lei, Ping Yan, Ai Shen, Wenbin Liu, Dewei Li, Zhongjun Wu

**Affiliations:** 1 The First Affiliated Hospital of Chongqing Medical University, No. 1 Youyi Road, Yuanjiagang, Yuzhong District, Chongqing, 400016 China; 2 Department of Hepatobiliary Pancreatic Tumor Center, Chongqing University Cancer Hospital, Chongqing, China; 3 CAS Key Laboratory of Infection and Immunity, CAS Center for Excellence in Biomacromolecules, Institute of Biophysics, University of Chinese Academy of Sciences, Chinese Academy of Sciences, Beijing, China

**Keywords:** TRIM37, Liver ischemia/reperfusion injury, TRAF6, IKKγ, Inflammation

## Abstract

**Background:**

Hepatic ischemia/reperfusion (I/R) injury is one of the major pathological processes associated with various liver surgeries. However, there is still a lack of strategies to protect against hepatic I/R injury because of the unknown underlying mechanism. The present study aimed to identify a potential strategy and provide a fundamental experimental basis for treating hepatic I/R injury.

**Method:**

A classic 70% ischemia/reperfusion injury was established. Immunoprecipitation was used to identify direct interactions between proteins. The expression of proteins from different subcellular localizations was detected by Western blotting. Cell translocation was directly observed by immunofluorescence. HE, TUNEL and ELISA were performed for function tests.

**Result:**

We report that tripartite motif containing 37 (TRIM37) aggravates hepatic I/R injury through the reinforcement of IKK-induced inflammation following dual patterns. Mechanistically, TRIM37 directly interacts with tumor necrosis factor receptor-associated factor 6 (TRAF6), inducing K63 ubiquitination and eventually leading to the phosphorylation of IKKβ. TRIM37 enhances the translocation of IKKγ, a regulatory subunit of the IKK complex, from the nucleus to the cytoplasm, thereby stabilizing the cytoplasmic IKK complex and prolonging the duration of inflammation. Inhibition of IKK rescued the function of TRIM37 in vivo and in vitro.

**Conclusion:**

Collectively, the present study discloses some potential function of TRIM37 in hepatic I/R injury. Targeting TRIM37 might be potential for treatment against hepatic I/R injury.Targeting TRIM37 might be a potential treatment strategy against hepatic I/R injury.

**Graphical Abstract:**

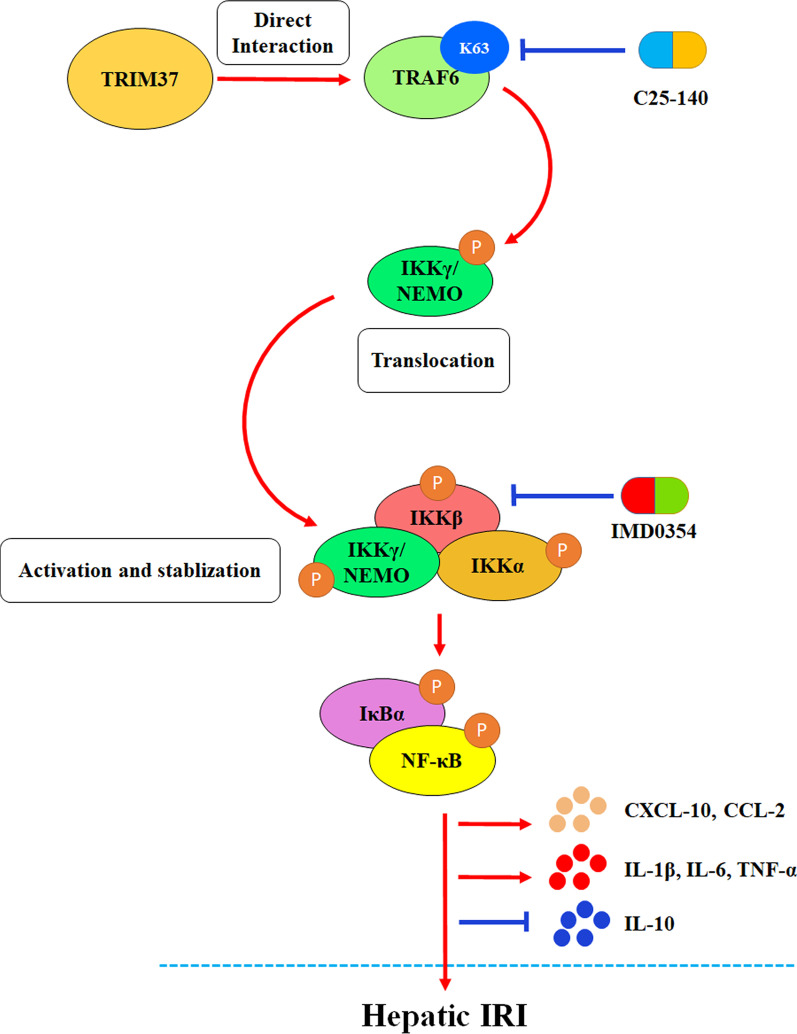

**Supplementary Information:**

The online version contains supplementary material available at 10.1186/s10020-023-00653-2.

## Introduction

Hepatic I/R injury is an inevitable pathological process associated with various liver operations (Ju et al. 2021). Oxidative stress and metabolic disorders in the early stage lead to the occurrence of robust inflammation that induces cell apoptosis and necrosis, ultimately resulting in liver dysfunction (Li et al. 2021a; Liu et al. 2019). Hyperactive inflammation acts as a bridge in these events. Kupffer cells are liver-resident macrophages and act as sentinels to expand the inflammatory signal toward parenchymal hepatic cells (Thorgersen et al. 2019; Wang et al. 2021a). Our previous results (Huang et al. 2020; Luo et al. 2021) and some other studies (Dar et al. 2019; Zhou et al. 2018) demonstrated that during this process, Kupffer cells are the receivers and transmitters, while hepatocytes are the main implementers in response to inflammatory signals derived from Kupffer cells. For instance, the inhibitor of nuclear factor kappa-B kinase (IKK) complex phosphorylates inhibitor of NF-κB (IκBα), causing sequential disintegration and activation of canonical IκBα/nuclear factor kappa B (NF-κB) in Kupffer cells. Phosphorylation and translocation of NF-κB p65 increases the secretion of inflammatory cytokines and chemokines, which induce stress and death in hepatocytes, as we previously explored (Huang et al. 2020; Li et al. 2021b). However, the specific mechanism of the IKK complex has not yet been fully disclosed because of the subunit diversity and functional complexity of IKK, which contains two catalytic subunits (IKKα and IKKβ) and one regulatory subunit (IKKγ).

Tumor necrosis factor receptor-associated factor 6 (TRAF6) is a pivotal E3 ubiquitin ligase associated with innate immunity in solid organs (Du et al. 2017; Xie et al. 2020). Unlike proteasome-dependent K48-chains, TRAF6-mediated nondegrading K63-chains lead to alterations in competence and the modification of substrates (Ni et al. 2019; Ohtake et al. 2016). In the liver, this conformational change can dramatically affect the transduction of downstream inflammatory signaling (Ben et al. 2019). We have previously identified TRAF6 as a vital mediator of external stress signals via K63 ubiquitination in liver I/R injury, which gives rise to the phosphorylation of TGF-beta activated kinase 1 (TAK1) and IKK activation (Huang et al. 2020, 2019; Liu et al. 2020). However, we failed to identify any molecule, either an E2 ligand or substrate, that might directly interact with TRAF6 during this process. Therefore, it is necessary to further explore the regulation of the IKK complex by TRAF6.

Tripartite motif containing 37 (TRIM37) is composed of a ring finger, a B-Box-type zinc finger, a B-Box C-terminal domain, a TRAF domain and a polyacid domain and has recently been reported to participate in a variety of inflammatory immune responses (Xiang et al. 2020; Chen et al. 2021a). Notably, evidence suggests that the inflammatory signals adjusted by TRIM37 might include MAPK (Zhu et al. 2020), mTOR (Wang et al. 2018) and NF-κB (Fu et al. 2021), which are common downstream targets of TRAF6. Although some characteristics of TRIM37 involve acting as an E3 ligand, other studies have also shown that TRIM37 plays other roles. In the present study, we investigated a novel role of TRIM37 in exacerbating hepatic I/R injury by directly interacting with TRAF6, thereby leading to the accumulation of TRAF6-linked K63 ubiquitin and subsequent IKK activation. Moreover, enrichment of TRIM37 could prolong the stability of the cytoplasmic IKK complex through the reinforcement of IKKγ translocation in a TRAF6-dependent manner. Taken together, the present study reveals the important function of TRIM37 in regulating the TRAF6/IKK axis in terms of intensity and phase, shedding some light on the mechanism and a therapeutic strategy for hepatic I/R injury.

## Materials and methods

### Cells

Primary Kupffer cells were isolated according to our improved extraction methods (Zhao et al. 2018; Pan et al. 2018). Briefly, in situ perfusion and digestion of the liver were performed using type IV collagenase. After low-speed separation of hepatic cells, a differential adherent protocol was used to isolate and purify primary Kupffer cells. Resuspended cells were cultured in F12/DMEM (1% dual antibiotics). A light rinse with PBS was performed 2 h after the inoculation. F4/80 was used as a classic marker of mature Kupffer cells.

### Hypoxia/reoxygenation (H/R) model for in vitro simulation

A standard hypoxia/reoxygenation (H/R) model was established according to our previous studies (Huang et al. 2020; Shen et al. 2020). Kupffer cells were seeded and cultured in a tri-gas incubator for hypoxia (1% O_2_, 94% N_2_ and 5% CO_2_, 6 h). After replacing the medium, a normal incubator was used for reoxygenation for different times (classic 24 h for conventional inflammatory markers, 1–24 h for subsequent stability evaluation of inflammatory protein). The cell density was approximately 70% before hypoxia treatment. Total RNA, supernatant and proteins were isolated after H/R treatment.

### Experimental animals

All animal procedures were approved by the Animal Care and Use Committee, Chongqing Medical University. All C57BL/6J mice in this study were 6–8 weeks old, male and standardly housed. A 12-h light/dark cycle was provided with free access to food and water. Stable temperature and humidity were strictly controlled. The mice weighed 24.1–28.5 g. Before treatment, all mice were randomly grouped.

### Hepatic ischemia/reperfusion (I/R) injury

Hepatic ischemia/reperfusion (I/R) injury was implemented based on our previous research (Huang et al. 2020; Mou et al. 2020; Chen et al. 2020). In brief, after anesthesia and midline abdominal opening, the intestine was separated and transferred to moist, room-temperature gauze for protection. After the exposure of vessels, the left and middle liver blood flow were prevented by a specific vessel clamp for 1 h of ischemia, followed by 12 h of reperfusion. Mice in the sham group underwent the same operation without vascular clipping. Serum and liver segments were collected immediately after reperfusion.

### Western blotting

Total protein was extracted using RIPA buffer (Beyotime, Shanghai, China) containing 10% cocktail protease inhibitor (Thermo, Waltham, USA). Cytoplasmic proteins and nuclear proteins were extracted using the ExKine Nuclear and Cytoplasmic Protein Extraction Kit (Abbkine, Beijing, China). β-Actin was used for the normalization of protein unless otherwise specified. Determination of TRIM37 (Proteintech, Wuhan, China), ASK1, p-ASK1, p-p38, p38, extracellular regulated protein kinases (ERK), p-ERK, p-JNK, JNK, c-caspase7, caspase7 (Zenbio, Chengdu, China), TRAF6, p-IKKα, p-IRAK1, IL-1β, IL-10, IKKγ, p-IKKγ (Abcam, Cambridge, UK), β-actin, p-IKK, IKK, IKKα, IKKβ, IL-6, TGF beta-activated kinase 1 (TAK1), p-TAK1, p-IκBα, IκBα, p-p65, p65, IRAK1, BAD, Bax, Bcl2, cleaved-caspase3 (c-caspase3), caspase3, Histone, α-tubulin (Beyotime, Shanghai, China), and p-IKKβ (Invitrogen, Carlsbad, USA) was accomplished as described previously (Huang et al. 2020, 2019; Pu et al. 2021). The dilution ratios of all primary antibodies are listed in Table 1.

**Table 1:** Antibody dilutions for Western blot

Antibody name	Dilution
TRAF6 (ab33915)	1/5000
IRAK1(ab180747), α-tubulin(ab7291)	1/3000
p-p65(AN371), p-IκBα(AF5851), IκBα(ab32518), TRIM37(ab264190), IKKγ(AG4319)	1/2500
β-actin(ab8226), IL-6(ab233706), p-ASK1(ab278547), p-ERK(ab131438), Histone(ab1791)	1/2000
JNK(ab199380), IKKβ(ab124957), c-Caspase3(ab32042), Caspase3(ab32351), IL-10(ab133575)	1/1500
p65(ab32536), p-IKKβ(ab194519), IKK(ab178870), p-IKK(ab194528), IKKα(ab32041), IL-1β(ab254360), p-TAK1(ab109404), TAK1(ab109526), ASK1(ab45178), p-p38(ab178867), ERK(ab32537), Bax(ab32503), Bcl2(ab32124), c-Caspase7(ab256469), Caspase7(ab255818), p-IKKγ(ab63551)	1/1000
p-JNK(ab47337), p38(ab170099), BAD(ab32445), p-IKKα(ab38515), p-IRAK1(PA5-105183)	1/500

### Assessment of liver pathologic changes and apoptosis

Typical pathological changes in hepatic I/R injury, including congestion, ballooning degeneration and necrosis, were observed using hematoxylin eosin (HE) staining based on our previous studies (Chen et al. 2021b; He et al. 2016). The standard Suzuki grade was used for quantification. Liver tissue was collected immediately after treatment. Immobilization was achieved with paraformaldehyde for 24 h at 4 ℃. Terminal deoxynucleotidyl transferase-mediated deoxyguanosine triphosphate (dUTP) nick-end labeling (TUNEL) staining was used to assess hepatic apoptosis. A commercial Roche in Situ Cell Death Detection Kit (Roche, Shanghai, China) was utilized for staining in accordance with the instruction manual. Image acquisition and normalization of HE and TUNEL staining were performed using ZEN2012 (Zeiss, Oberkochen, Germany).

### Cytokines and liver enzymes

Cytokines in serum and supernatant were detected using commercial enzyme-linked immunosorbent assay kits (Beyotime) to determine local or systemic inflammatory conditions. Liver enzymes in serum, including alanine aminotransferase (ALT) and aspartate aminotransferase (AST), were detected by microplate kits (JianCheng Bioengineering Institute, Nanjing, China) to determine liver function. The number of wells for each indicator was at least 3 and was specifically denoted in the relevant figure legend.

### Quantitative real-time PCR (qRT‒PCR)

TRIzol reagent (Beyotime) was used to isolate RNA from cells or tissues based on our previous research (Huang et al. 2020; Li et al. 2021b). The relative expression of CXC chemokine ligand 10 (CXCL-10), C–C Motif Chemokine ligand 2 (MCP1, CCL-2), CXCL-2 and IL-10 was normalized to β-actin. All primer sequences are provided in Table 2. The relative expression was calculated using the 2^−ΔΔCt^ method. The number of wells for each indicator was at least 3 and was specifically denoted in the relevant figure legend.

**Table 2:** Sequence

Name	Sequence
IL-10 forward	AACCCAGGCACATCCGAAAAGC
IL-10 reverse	AGAGACTACGCAGAGACCACAGAC
CXCL-2 forward	ATGCCTGAAGACCCTGCCAAG
CXCL-2 reverse	GGTCAGTTAGCCTTGCCTTTG
MCP-1 forward	TTTTTGTCACCAAGCTCAAGAG
MCP-1 reverse	TTCTGATCTCATTTGGTTCCGA
CXCL-10 forward	CAACTGCATCCATATCGATGAC
CXCL-10 reverse	GATTCCGGATTCAGACATCTCT
HRPT forward	TCAACGGGGGACATAAAAGT
HRPT reverse	TGCATTGTTTTACCAGTGTCAA
β-actin forward	GGCTGTATTCCCCTCCATCG
β-actin reverse	CCAGTTGGTAACAATGCCATGT

### Transfection and administration in vitro

Adenovirus containing sh-TRIM37 was transfected into cells for 48 h before treatment, and the transfection efficiency was verified. The first fluid renewal was performed 6 h after transfection. Transfection of si-TRAF2, si-TRAF4 and si-TRAF6 was accomplished using Lipo6000™ Transfection Reagent (Beyotime) 48 h before treatment. All interference sequences are listed in Table 3. C25-140, the specific inhibitor of the TRAF6-Ubc13 interaction, was administered 2 h before subsequent treatment with 20 μM based on our previous studies (Huang et al. 2020; Luo et al. 2021; Li et al. 2021b). The specific IKKβ inhibitor IMD0354 was administered 12 h before subsequent treatment with 10 μM based on our previous studies (Huang et al. 2020; Luo et al. 2021; Li et al. 2021b).

**Table 3:** Sequence

Name	Sequence
sh-TRIM37	AAGCTGCACAGACTGGTTATA
si-TRAF2	GGGUCUUCAUCUGGAAGAUTT
	AUCUUCCAGAUGAAGACCCTT
si-TRAF4	CAGCCUUCUAUACGCAUAATT
	UUAUGCGUAUAGAAGGCUGTT
si-TRAF6	GCCCAGUUGACAAUGAAAUTT
	AUUUCAUUGUCAACUGGGCTT

### Transfection and administration in vivo

Adenovirus containing sh-TRIM37 was transfected in vivo (8 × 10^8^ pfu, tail vein) 7 days before treatment. C25-140 was administered (14 mg/kg, intraperitoneal injection) at 24 h and 12 h before the treatment based on our previous research (Huang et al. 2020; Luo et al. 2021; Li et al. 2021b). IMD0354 was administered (5 mg/kg, intraperitoneal injection) 12 h before the treatment based on our previous research (Huang et al. 2020; Luo et al. 2021; Li et al. 2021b).

### Immunofluorescence and coimmunoprecipitation

Cells were spread evenly onto glass slides for immunofluorescence analysis. After the corresponding treatment, immobilization was completed using paraformaldehyde. Blockade and permeation were accomplished using Triton X-100 (0.3%) dissolved in QuickBlock™ Blocking Buffer for Immunol Staining (Beyotime) for 20 min. The primary antibodies were as follows: F4/80 (Abcam, 1: 100), p65 and p-IKKβ (Beyotime, 1: 100), TRIM37 (Proteintech, 1: 150), p-IKKγ (Abcam, 1: 100), TRAF6 (Beyotime, 1: 250), and IKK (Beyotime, 1: 200). F4/80 was used as the marker of functional Kupffer cells. All primary antibodies were incubated for 8 h in a 4 °C room. Secondary antibodies conjugated with Cy3 or FITC were incubated at 37 °C for 30 min, followed by nuclear staining with DAPI (Beyotime). Cells were lysed after being treated using a specific lysis buffer containing HEPES (25 mmol/L), NaCl (150 mmol/L), EDTA (1 mmol/L), 2% glycerol and PMSF (1 mmol/L) based on previous studies (Liu et al. 2020; Wu et al. 2018). Samples were oscillated repeatedly and placed on ice for 30 min. After centrifugation and preclearing, incubation with antibody-cross-linked protein G-agarose beads was performed for 8 h in a 4 °C room. Immunoblot analysis was then performed after pipetting and resuspending-premix.

### Statistical analysis

The LSD-t test was used for comparisons of results between 2 groups. Welch’s correction was performed if there was any heterogeneity of variance. One-way analysis of variance (ANOVA) followed by Bonferroni’s post hoc test was used for comparisons among multiple groups. In vitro repetition was at least 3 and is described in detail in the corresponding figure legend. Sample sizes in vivo are shown in the corresponding figure legend. P < 0.05 was considered to be statistically significant. Statistical calculations were analyzed using SPSS (version 19.0).

## Results

### Inhibition of TRIM37 relieved hepatic I/R injury

**Fig. 1:**
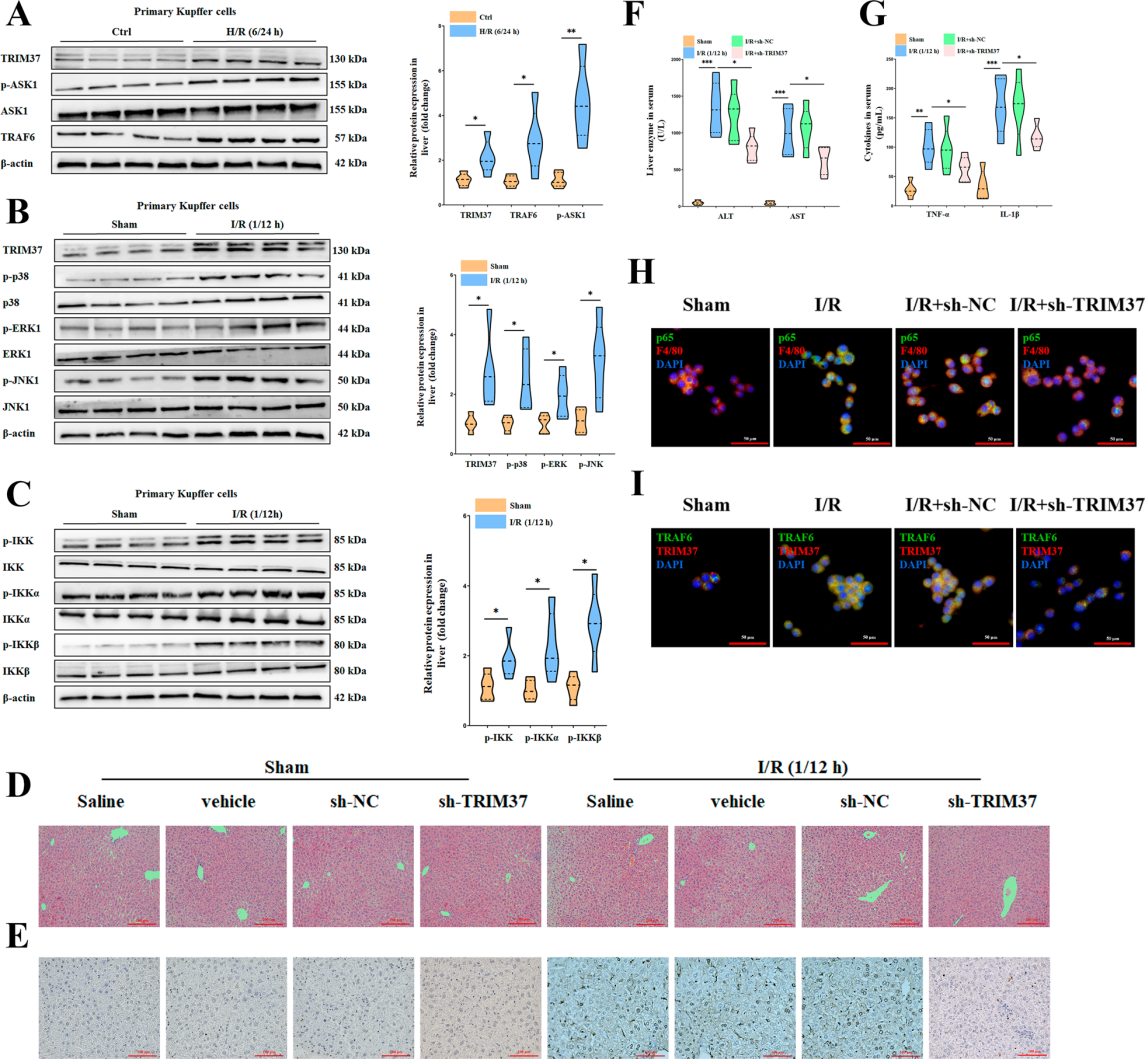
Inhibition of TRIM37 relieved hepatic I/R injury. **A** TRIM37 expression was upregulated in vitro. **B**, **C** Protein expression of the TRIM37, MAPK and IKK pathways was increased in vivo. **D**, **E** Interference of TRIM37 alleviated hepatic pathological injury and apoptosis in hepatic I/R injury. Scale bar = 200 μm for HE, scale bar = 100 μm for TUNEL. **F**, **G** Liver enzymes and cytokines in mouse serum after inhibiting TRIM37 in hepatic I/R injury. **H**,** I** Immunofluorescence analysis of p65, F4/80, TRAF6 and TRIM37 in primary Kupffer cells after hepatic I/R injury. Scale bar = 50 μm. *p < 0.05, **p < 0.01, ***p < 0.001, ^N.S^*p* > 0.05, n = 5–6 for each group, the two lanes in a row are from the same mixed samples

We identified the expression of TRIM37 and classical inflammatory signaling proteins in hepatic I/R injury in vivo and in vitro. H/R insult stimulated TRIM37 expression in Kupffer cells and increased the levels of p-ASK1 and TRAF6 (Fig. 1A, B). Analogously, I/R exposure led to phosphorylation of ERK1 and IKK (Fig. 1B, C). Hence, we intervened with TRIM37 in vivo for further examination. Hepatic pathological injury and apoptosis caused by I/R were alleviated by TRIM37 deficiency, as expected (Fig. 1D, E, Suzuki scores are presented in Additional file 1: Fig. S1A). Inhibition of TRIM37 could reduce liver enzymes and cytokines in serum (Fig. 1F–G). Activation of NF-κB p65 and TRAF6 in primary Kupffer cells derived from I/R-treated livers was restrained by the downregulation of TRIM37 (Fig. 1H, I), suggesting a latent contribution of TRIM37 to hepatic I/R injury.

### TRIM37 aggravated liver I/R injury via the reinforcement of IKK but not ASK1/MAPK

**Fig. 2:**
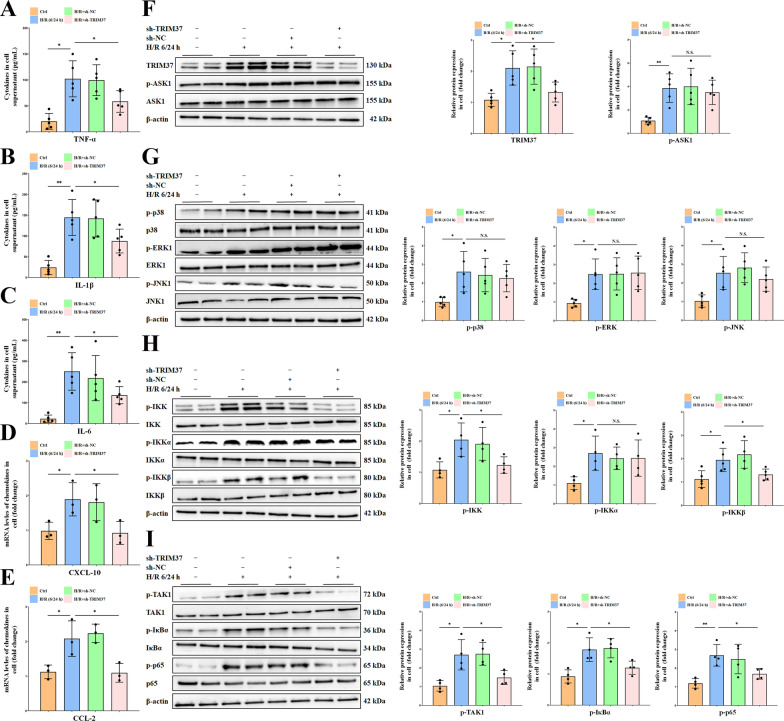
TRIM37 reinforced IKK instead of ASK1/MAPK. **A**–**E** TRIM37 knockdown augmented the expression of cytokines and chemokines in primary Kupffer cells after H/R treatment. **F** Inhibition of TRIM37 weakened IL-6 but not p-ASK1 protein expression. **G** MAPK pathway proteins were hardly altered by TRIM37 deficiency. **H** The IKKβ/IKK complex was significantly suppressed when TRIM37 expression was restricted in vitro. **I** The phosphorylation of TAK1/IκBα/NF-κB p65 was reduced by TRIM37 repression. Two lanes in a row are from the same mixed samples. All in vitro experiments were repeated at least 3 times. *p < 0.05, **p < 0.01, ^N.S^*p* > 0.05

Although IKK and MAPK showed some correlation with TRIM37, it has not yet been determined whether these signals are involved in the effect of TRIM37 on alleviating hepatic inflammation. The protein analysis demonstrated that TRIM37 promoted the upstream IKK complex, while the ASK1/MAPK pathway was insignificantly affected (Fig. 2A–I). We then focused on the direct upstream factor of the IKK complex and found that the phosphorylation of TAK1 was inhibited after suppressing TRIM37 (Fig. 2F). These results suggested that TRIM37-mediated inflammatory progression in the liver might depend on the IKK complex instead of the ASK1/MAPK pathway.

### Enrichment of TRIM37 exacerbated hepatic I/R injury

**Fig. 3:**
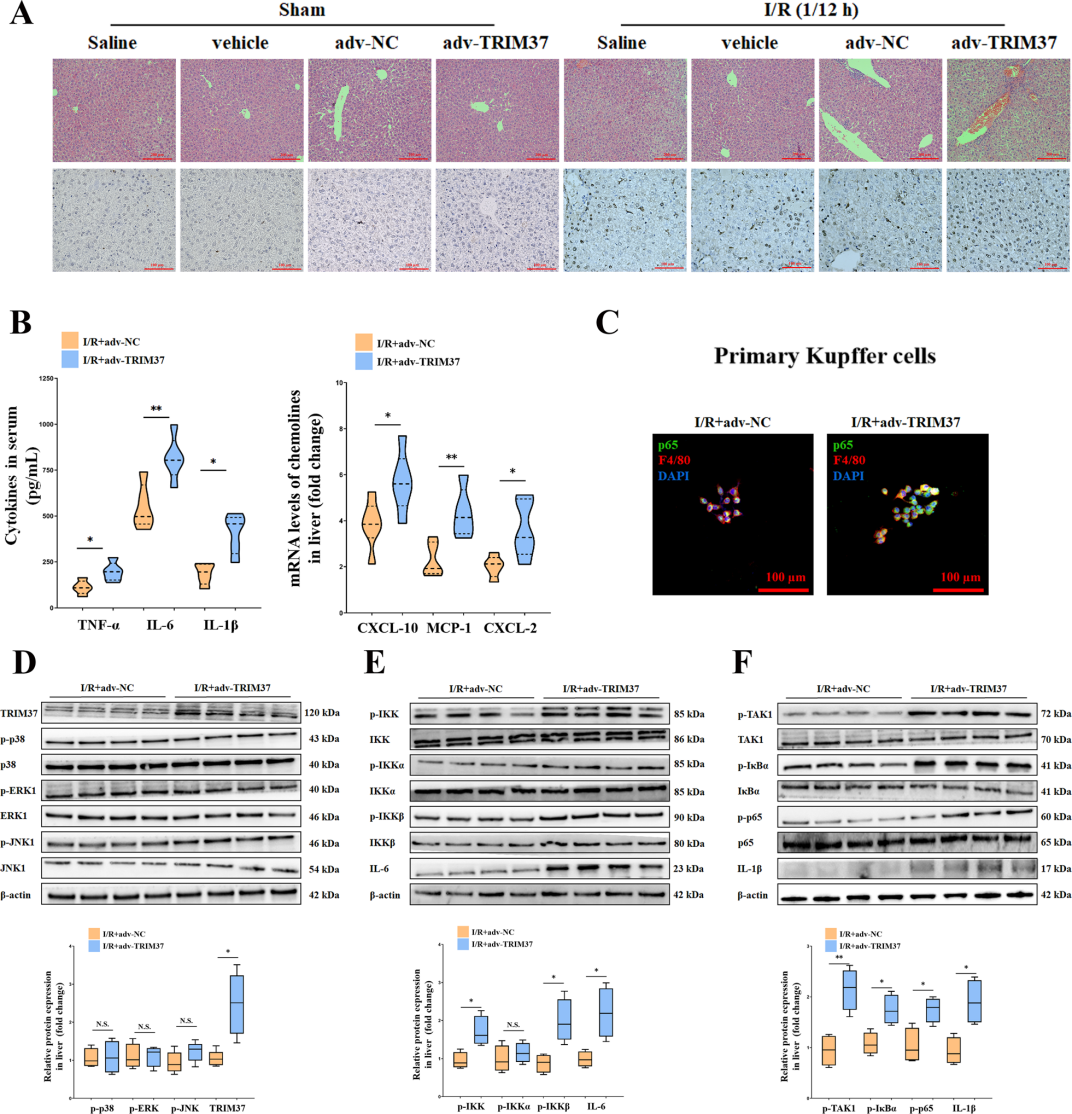
Enrichment of TRIM37 exacerbated hepatic I/R injury. **A** Overexpression of TRIM37 aggravated hepatic pathological injury and apoptosis in vivo. Scale bar = 200 μm for HE, scale bar = 100 μm for TUNEL. **B** TRIM37 increased serum cytokines and chemokines in mice. **C** Immunofluorescence analysis of p65 and F4/80 in primary Kupffer cells after I/R treatment. Scale bar = 50 μm for immunofluorescence. **D** Protein expression of TRIM37 and the MAPK pathway after TRIM37 enrichment in vivo. **E** Protein expression of the IKK pathway after TRIM37 enrichment in vivo. **F** The activation of TAK1/IκBα/NF-κB p65 after TRIM37 enrichment in vivo. *p < 0.05, **p < 0.01, ***p < 0.001, the two lanes in a row are from the same mixed samples, n = 5–6 for each group

To further determine the role of TRIM37, we investigated the effect of TRIM37 overexpression on hepatic I/R injury. As shown in Fig. 3A, over-expression of TRIM37 resulted in more severe congestion, steatosis, ballooning (above, Additional file 1: Fig. S1B) and apoptosis (below) in the liver, indicating that an increase in TRIM37 deteriorated hepatic pathological lesions and apoptosis. Likewise, cytokines and chemokines were increased by TRIM37, resulting in a more severe inflammatory condition (Fig. 3B). Immunofluorescence showed that the IKK complex was strengthened when TRIM37 was upregulated in primary Kupffer cells in vivo (Fig. 3C). The Western blot results confirmed the hyperactivation of TAK1/IKKβ (Fig. 3D–F).

### TRIM37 accelerated K63 ubiquitination by directly binding to TRAF6

**Fig. 4:**
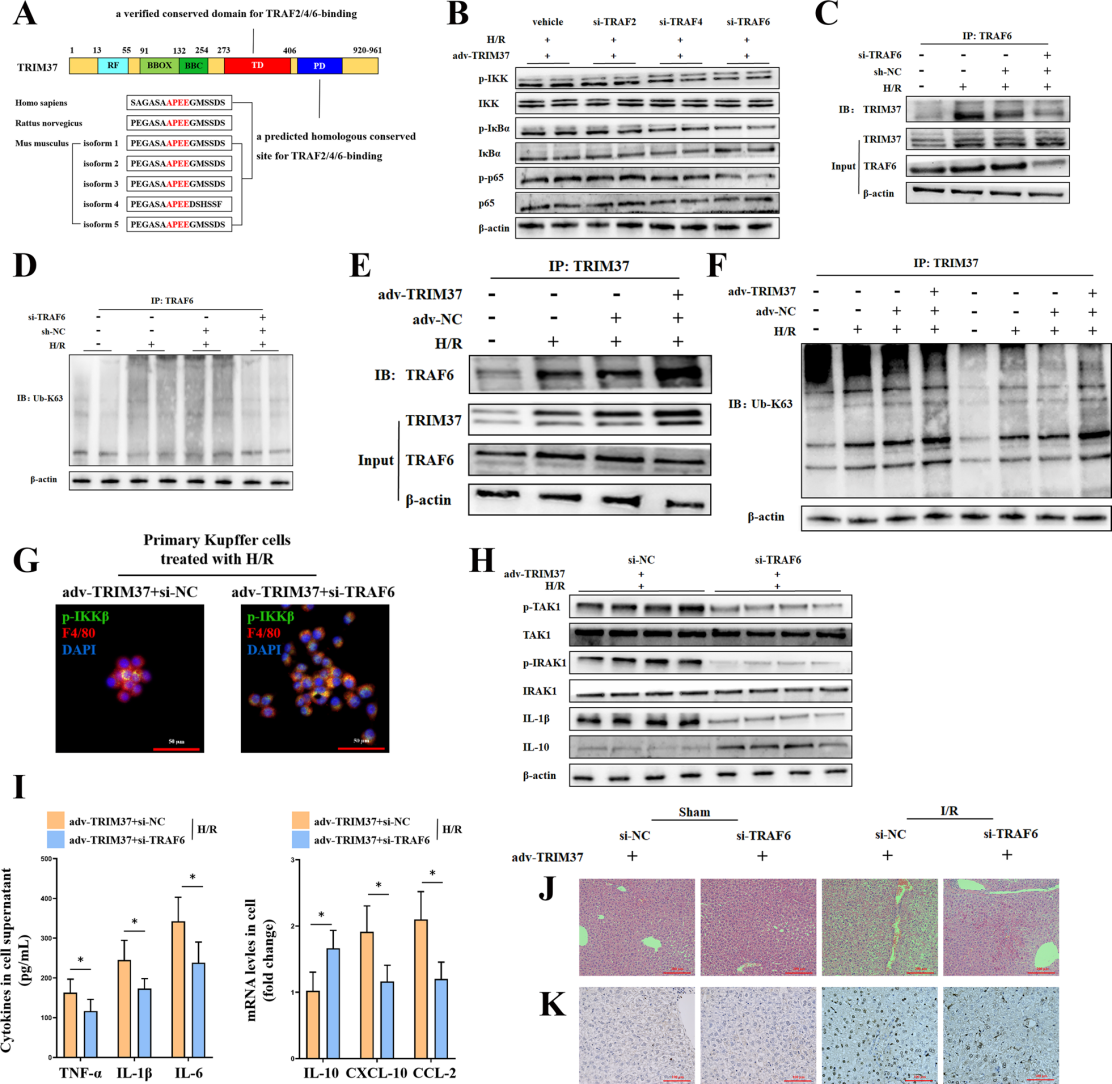
TRIM37 increased the K63 chain by directly binding to TRAF6. **A** Alignment of multispecies homologous amino acid sequences of TRIM37 and its conserved domains. **B** Effectiveness after the inhibition of TRAF2, TRAF4 and TRAF6 in vitro. **C** The interaction between TRAF6 and TRIM37 was detected by immunoprecipitation after TRAF6 inhibition in vitro. **D** Determination of TRAF6-guided K63 ubiquitin. **E** The interaction between TRAF6 and TRIM37 was detected by immunoprecipitation after TRIM37 overexpression in vitro. **F** Determination of TRAF6-guided K63 ubiquitin in the presence of TRIM37 overexpression. **G** Immunofluorescence analysis of p-IKKβ and F4/80 in primary Kupffer cells after H/R exposure, scale bar = 50 μm for immunofluorescence. **H** Protein expression of TAK1 when TRAF6 was suppressed in vitro. **I** Cell cytokines and chemokines after suppressing TRAF6. **J**, **K** The lack of TRAF6 inhibited hepatic pathological changes and apoptosis in liver I/R injury under the condition of TRIM37 upregulation. Scale bar = 200 μm for HE, scale bar = 100 μm for TUNEL. *p < 0.05

To further demonstrate the mechanism through which TRIM37 facilitates IKK inflammatory signaling, we analyzed conserved homologous sequences and domains of TRIM37 among common species (Fig. 4A). There is a verified TRAF domain (TD) within a predicted site in the polyacid domain (PD) containing a specific alignment of alanine/proline/glutamic acid/glutamic acid (A/P/E/E) (Wu et al. 2018), which might have the ability to bind TRAF2 (Fig. 4A). Since the binding motif of TRAF2 has probable universality among multiple members of several TRAF family members (including TRAF2/4/6), we verified their functions (Fig. 4B). TRAF6 seems to be mostly involved in this process (Fig. 4B). Because TRAF6 itself is an important E3 ligand, we then examined whether there was a direct interaction between TRIM37 and TRAF6 in this process. As shown in Fig. 4C–F, TRIM37 directly binds to TRAF6, thereby promoting its K63 ubiquitin. Moreover, interference of TRAF6 was able to abolish the function of TRIM37 in vitro (Fig. 4G–I) and in vivo (Fig. 4J–K, Additional file 1: Fig. S1C). These results suggest that TRIM37 aggravates hepatic I/R injury in a TRAF6-dependent manner.

### Downregulation of TRAF6 abrogated TRIM37-induced hepatic inflammation

**Fig. 5:**
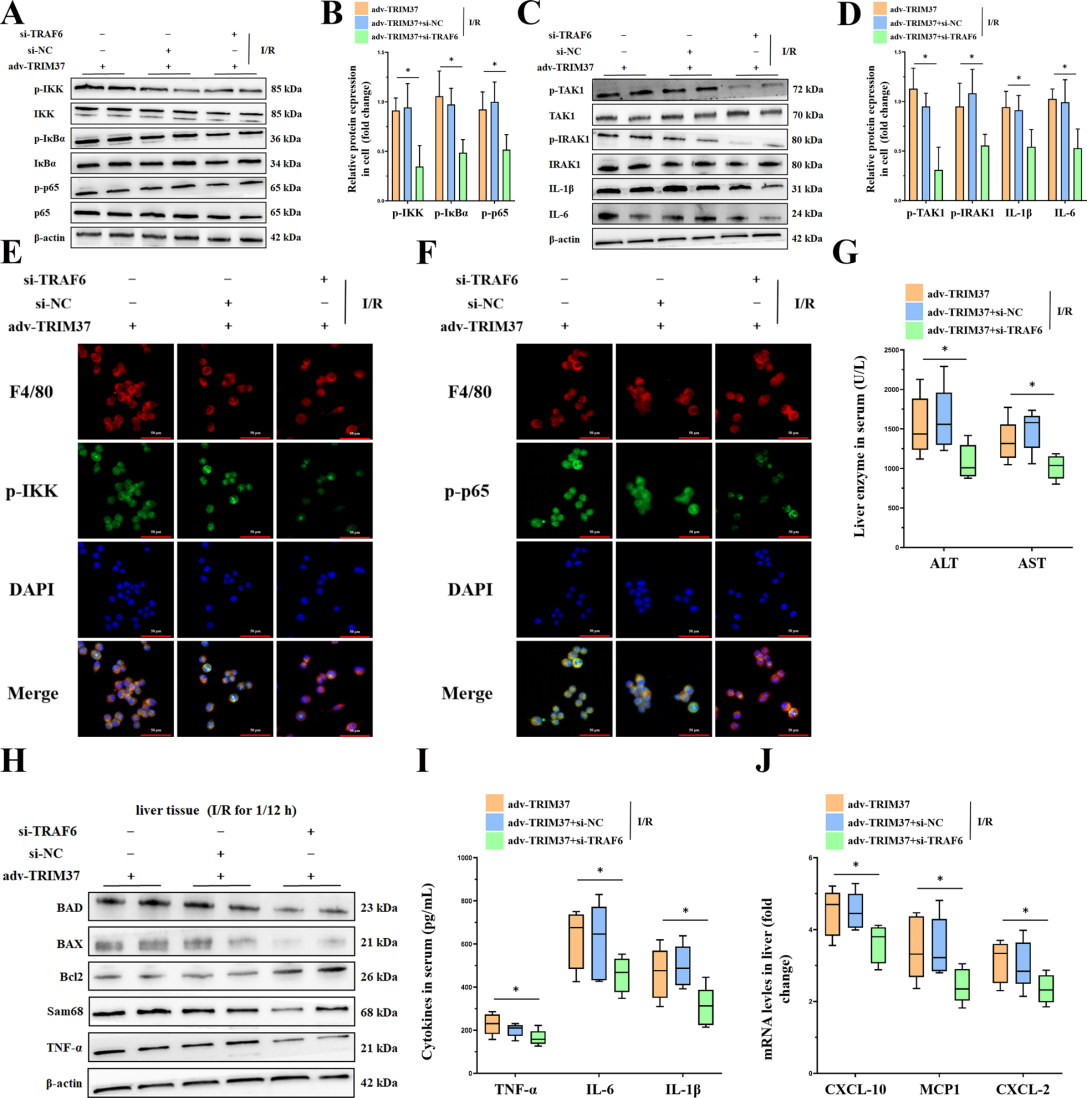
Downregulation of TRAF6 abrogated TRIM37-induced hepatic inflammation. **A**, **B** Protein expression of the IKK pathway after interfering with TRAF6. **C**, **D** Protein expression of TAK1 after interfering with TRAF6. **E**, **F** Immunofluorescence analysis of p-IKK, p-p65 and F4/80 in primary Kupffer cells derived from I/R-treated liver, scale bar = 50 μm for immunofluorescence. **G** Liver enzymes in mouse serum after TRAF6 suppression. **H** The expression of apoptosis-related proteins in liver tissue after restraining TRAF6 in vivo. **I**, **J** Cytokines and chemokines in mice. ^N.S^*p* > 0.05, *p < 0.05, **p < 0.01. Two lanes in a row are from the same mixed samples, n = 5–6 for each group

Consistent with the pathologic alterations, disturbances in TRAF6 impaired protein expression of the TAK1/IKK pathway induced by TRIM37 augmentation (Fig. 5A–D). Immunofluorescence indicated attenuated activity of NF-κB and IKK in response to TRAF6 deficiency (Fig. 5E, F). Similarly, serum enzymes, including ALT and AST, were decreased when TRAF6 was suppressed by TRIM37 enhancement (Fig. 5G). We further evaluated liver outcomes by assessing apoptosis-related proteins. As predicted, the expression of apoptosis-related proteins was weakened in the si-TRAF6 group (Fig. 5H). Likewise, the release of cytokines and chemokines resulting from TRIM37 overexpression was decreased by TRAF6 inhibition (Fig. 5I, J).

### Specific inhibition of the TRAF6-Ubc13 interaction partially rescued hepatic I/R injury during TRIM37 amplification

**Fig. 6:**
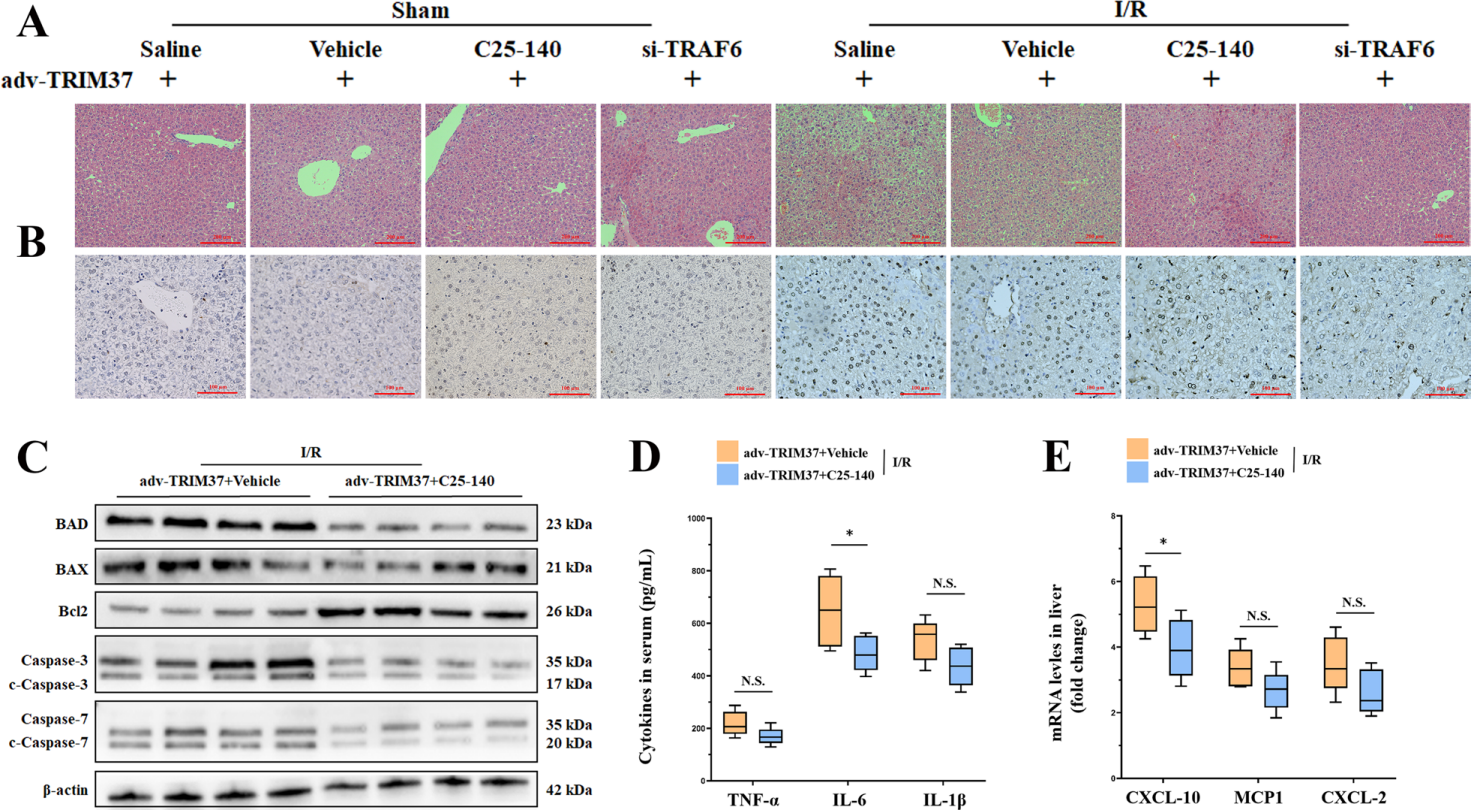
Inhibition of the TRAF6-induced K63 chain only partially rescued hepatic I/R injury under TRIM37 amplification. **A**, **B** Hepatic pathological changes and apoptosis after C25-140 treatment or TRAF6 inhibition. Scale bar = 200 μm for HE, scale bar = 100 μm for TUNEL. **C** The expression of apoptosis-related proteins in liver tissue after restraining C25-140 utilization in vivo. **D** Levels of cytokines in mouse serum. **E** Levels of chemokines in the liver. ^N.S^*p* > 0.05, ^*^p < 0.05. Two lanes in a row are from the same mixed samples, n = 5–6 for each group

To explore the relevant mechanism in depth, we used C25-140, a specific inhibitor of the TRAF6-Ubc13 interaction. Surprisingly, despite some selective benefits, C25-140 failed to completely relieve the aggravation of liver damage caused by TRIM37 (Fig. 6A, B; Additional file 1: Fig. S1D). Apoptosis-related proteins were significantly lower in the C25-140 group, indicating that there was still a positive effect on hepatocytes (Fig. 6C). Moreover, there was limited downregulation of cytokines and chemokines, suggesting that the control of local inflammation by C25-140 might not be thorough (Fig. 6D–E).

### TRAF6 prolonged the inflammatory phase by stabilizing the IKK complex in hepatic I/R injury

**Fig. 7:**
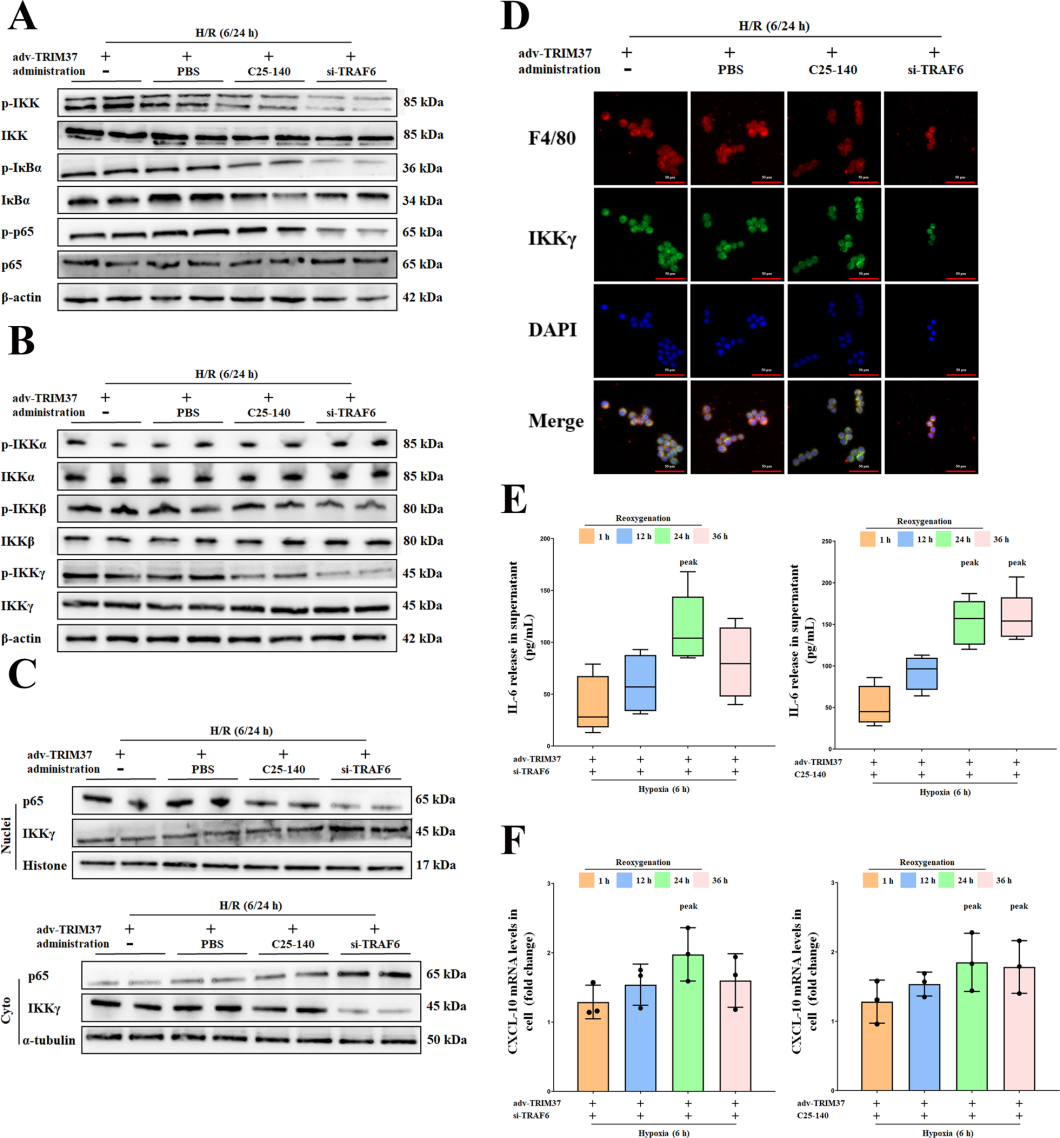
TRAF6 prolonged the inflammatory phase by stabilizing the IKK complex in vitro. **A** Inhibition of TRAF6 in vitro has a stronger anti-inflammatory effect than ubiquitin repression by C25-140. The protein expression of IKK/IκBα/NF-κB was detected at 6/24 h after H/R. **B** The protein expression of IKK catalytic and regulatory subunits was detected at 6/24 h after H/R. **C** The translocation of IKKγ was monitored at 6/24 h after H/R. **D** Immunofluorescence analysis of IKKγ and F4/80 in primary Kupffer cells after H/R treatment, scale bar = 50 μm for immunofluorescence. **E**, **F** Changes in the expression of IL-10 and CXCL-10 in the cell supernatant from 1 to 36 h after reoxygenation with TRIM37 amplification and TRAF6 deficiency. Two lanes in a row are from the same mixed samples, the in vitro experiment was repeated independently at least 3 times

In consideration of the limited benefits of C25-140, which was not as ideal as expected, we next explored whether its effect on the downstream inflammatory signaling pathway was consistent with widespread interference with TRAF6. At 6/24 h after H/R, the efficacy of C25-140 was not as good as the widespread downregulation of TRAF6 (Fig. 7A). Further detection of the IKK complex showed that IKKγ was the subunit that was most sensitive to the extensive inhibition of TRAF6 (Fig. 7B). Considering that our previous focus was limited to the catalytic subunit of the IKK complex, we hypothesized that the regulatory subunit IKKγ might participate. IKKγ promotes nonclassical IKK/NF-κB, which is achieved through specific nuclear-cytoplasmic translocation. As demonstrated in Fig. 7C, D, translocation of IKKγ could be reversed by TRAF6 deficiency but not C25-140 when TRIM37 was overexpressed. We then explored the broader phase of reoxygenation. Notably, although these treatments had significant anti-inflammatory effects on the early stages (1–36 h), the downregulation of TRAF6 prematurely ended the 24-h inflammatory peak, while C25-140 did not (Fig. 7E–F). These results suggested that in addition to acting as an E3 ligand, TRAF6 could prolong the duration of TRIM37-induced inflammation by promoting IKKγ translocation.

### Inhibition of IKKβ abolishes the effectiveness of IKKγ translocation

**Fig. 8:**
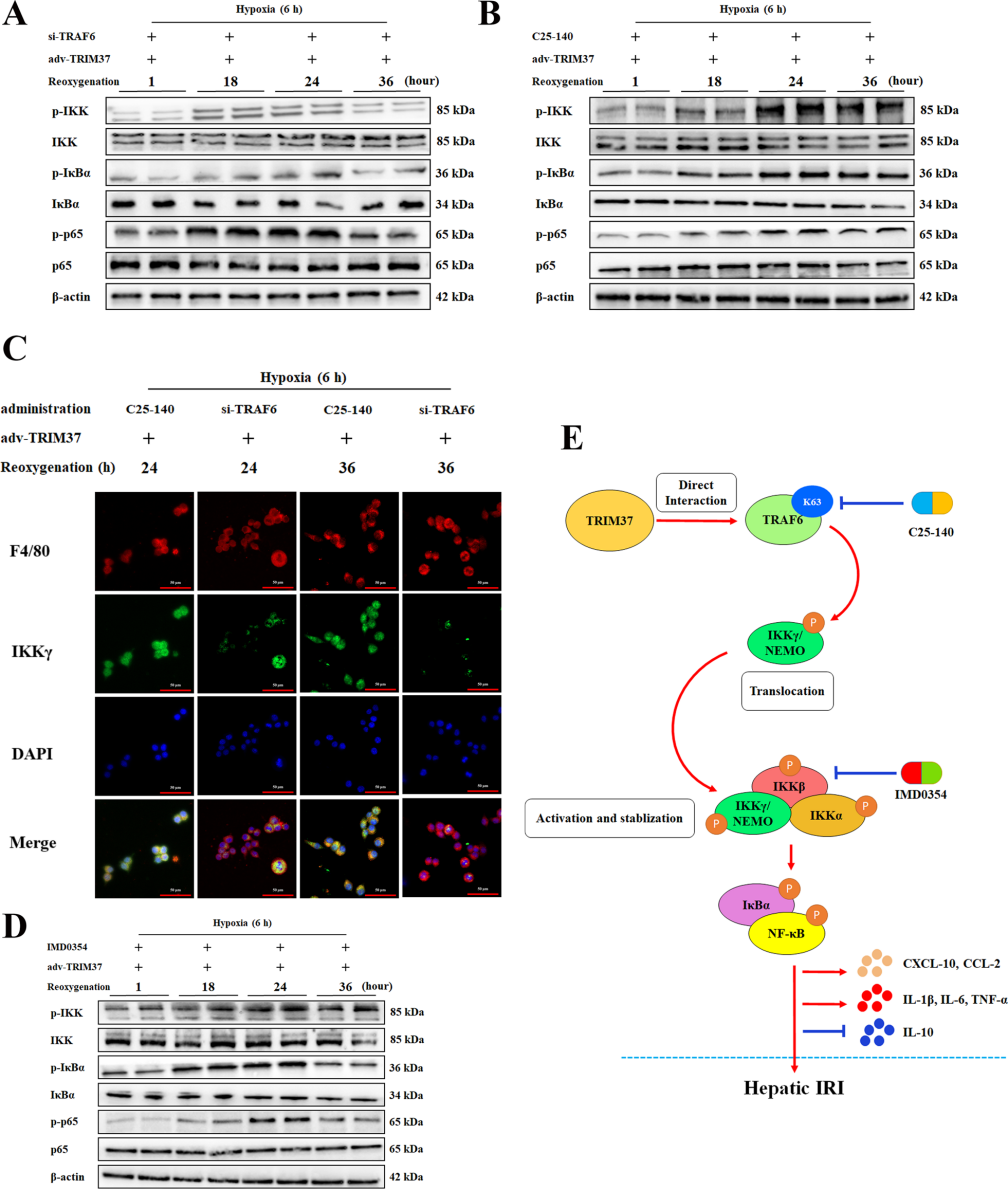
Inhibition of IKKβ abolished the effectiveness of IKKγ translocation. **A**, **B** The influence of C25-140 and TRAF6 the inhibition on IKK/IκBα/NF-κB proteins was detected when TRIM37 was overexpressed after 1 h to 36 h of reoxygenation. TRAF6 inhibition prematurely ended the 36-h spike in inflammation, but C25-140 did not. **C** Immunofluorescence analysis of IKKγ and F4/80 in primary Kupffer cells after H/R treatment and 24 h and 36 h of reoxygenation, scale bar = 50 μm for immunofluorescence. **D** The specific IKKβ inhibitor IMD0354 was used for further study. The influence of IMD0354 on IKK/IκBα/NF-κB protein was detected. **E** The underlying mechanism by which TRIM37 aggravates liver I/R injury through TRAF6 ubiquitination and IKKγ translocation. The activation of IKK inflammatory signaling pathway was induced by the activation of K63 ubiquitination derived from the binding of TRIM37 to TRAF6. Cytoplasmic translocation of IKKγ might be one of the main functional IKK subunits in this process. Subsequently, canonical NF-κB p65 is phosphorylated and transported into the nucleus, resulting in the release of inflammatory cytokines and chemokines. TRIM37: tripartite motif containing 37. TRAF6: tumor necrosis factor receptor-associated factor 6. IKK: inhibitor of nuclear factor kappa-B kinase. IκBα: inhibitor of NF-κB. NF-κB: nuclear factor kappa B. CXCL-10: CXC chemokine ligand 10. CCL-2: C–C Motif Chemokine ligand 2. IRI: ischemia/reperfusion injury. Two lanes in a row are from the same mixed samples, the in vitro experiment was repeated independently at least 3 times.

We then explored whether IKKγ affected hepatic I/R injury independently of IKKβ, partially or in whole. Western blot analysis of the IKK pathway indicated that interference with TRAF6 reduced the maintenance phase of inflammation from 36 to 24 h (Fig. 8A, B). IKKγ translocation from the nucleus to the cytoplasm was inhibited by TRAF6 inhibition at different time points (Fig. 8C). However, IMD0354, as a specific IKKβ inhibitor, decreased IKK excitation when TRIM37 was uptaken in both intensity and phase (Fig. 8D). These functional tests confirmed the protective effect of IKKβ depletion against TRIM37/TRAF6-driven hepatic I/R injury in vivo (Fig. 8E).

## Discussion

There are few strategies for the diagnosis or treatment of liver I/R injury due to its mechanical complexity, which includes oxidative stress, metabolic disorders, uncontrolled inflammation, apoptosis, and necrosis at diverse stages (Wang et al. 2019, 2021b). Kupffer cells, which are the primary immune cells in the liver, relay and amplify hepatic inflammatory signals, thereby driving parenchymal cells to functional disorders in the absence of effective interventions (Li et al. 2020a; Lu et al. 2018). TAK1 promotes the transduction of the pivotal inflammatory signals IKK and MAPK in hepatic I/R injury (Chen 2012; Guo et al. 2020). Nevertheless, due to upstream diversity, TAK1 exhibits selective activation. The present study demonstrated that TRIM37 accelerated the phosphorylation of TAK1 via direct interactions with TRAF6. Importantly, this binding strengthened and prolonged activation of the IKK complex instead of the MAPK pathway in hepatic I/R injury.

We have previously discussed that TRAF6, a direct upstream target of TAK1, promotes liver inflammation during hepatic I/R injury by mediating inflammatory signals in immune cells and apoptotic signals in parenchymal cells (Luo et al. 2021; Li et al. 2021b, 2020b; Liu et al. 2020; Huang et al. 2019). By binding to the E2 ligand, TRAF6 acts as an E3 ligand to guide the K63-ubiquitination of various substrate proteins. Although we and other researchers have previously demonstrated the potential role of TRAF6 in hepatic IRI, the latent interacting molecules with TRAF6 in this model remain unclear for us. Through protein interaction and post-translational modification, inflammatory signals including IKK pathway are activated, and finally inflammatory cytokines (such as TNF-α, IL-1β, IL-6) and chemokines (such as CXCL-10, CCL2) are released. These cascades alter the microenvironment of the liver, increase inflammatory progression, and impair liver function.

Some common endogenous microRNAs (miRs) (such as miR-125) can directly target TRAF6 and lead to its overall expression, as we verified (Huang et al. 2019). Moreover, regulation of TRAF6 at the modification level has a certain effect (Li et al. 2021b, 2020b; Liu et al. 2020). For instance, we previously showed that F-box/WD repeat-containing protein 5 (FBXW5), a newly discovered E3 ligand of apoptotic signal-regulating kinase 1 (ASK1), promoted the TRAF6-K63 chain by enhancing the phosphorylation of ASK1 (Li et al. 2021b). Likewise, other research reported that TRAF6 ubiquitinating activity is required for toll-interacting protein (Tollip) regulation of the ASK1-MAPK axis in liver I/R injury (Yan et al. 2019). Herein, the present study revealed a novel role of TRAF6 in TRIM37-guided activation and stabilization of the IKK complex. The direct binding of TRAF6 to TRIM37 is required for TRAF6-K63 ubiquitin and the phosphorylation of TAK1, which could be significantly reversed by TRAF6-Ubc13 inhibition. This results in a series of phosphoric group transfers, eventually leading to the activation of IKKβ and the unbinding of IκBα/p65. Moreover, the interaction between TRAF6 and TRIM37 is necessary for the cytoplasmic translocation of IKKγ, which consequently stabilizes and prolongs the IKK complex. Notably, alterations in of overall expression of TRAF6 rather than changes in its E3 activity could influence this step more effectively. The limited benefit from C25-140 and its low efficacy against IKKγ translocation suggested that the involvement of TRAF6 in the TRIM37-guided NF-κB cascade might not entirely depend on the link of K63. However, due to the difficulty of endogenous enrichment and detection, we thus far have failed to explore a triple combination. In fact, the nonclassical IKK maintenance exhibited by IKKγ cytoplasmic transfer is not rare. During genetic stress, IKKγ translocation from the nucleus to the cytoplasm is essential for the maintenance of high IKK complex activity (Wu et al. 2018). Moreover, the slight reduction promoted by IMD0354 at 36 h in p-p65 and p-IκBα should be quantified by densitometry, this is another limitation of the present study. Considering that the p-IκBα signal is more reduced in si-TRAF6, combination of the IKKβ inhibition and si-TRAF6 to reduce the NF-κB activation might be a potential strategy.

The functional diversity of TRIM37 includes its participation in disparate conditions (Avela et al. 2000; Meitinger et al. 2020). In the embryonic stage, mutation of TRIM37 leads to developmental disorders in a variety of solid organs (Avela et al. 2000). The ability to regulate organ development, organelle homeostasis and tumorigenesis is the main characteristic of TRIM37, drawing concern in the first two decades. TRIM37 has a binding domain for TRAF2/4/6, although this site shows different tendencies in different situations. Moreover, our results indicate that there is a predicted site with the order of P/A/S/T-X-Q/E‒E, which might be another locus for TRAF binding. As for immunofluorescence in the present study, co-localization is sometimes a difficult phenomenon to demonstrate through 2-D immunofluorescence, especially in cells in which the nuclei take up the majority of the cell architecture. Perhaps 3-D imaging with subsequent detailed mathematical co-localization analysis would be more appropriate.

It has been shown that TRIM37 affects cell proliferation and is highly expressed during mitosis in other cells (Brigant et al. 2020). In the present study, we only paid attention to the inflammatory indicators in the acute stage of liver IRI and the early pathological injury of liver, but did not evaluate the effect of TRIM37 on liver cell proliferation. This is one of the limitations of this study. The proposed site in the present study is only one of the potential predictive binding sites, since experiments of site mutations have not been tested. Some other sites seem to be also latent predicted motif allowing TRAF6 binding using different tools. At present, we have preliminarily demonstrated their functions in hepatic IRI, and conducted preliminary reasoning and observation on the potential mechanism of action, which is our shortcoming.

## Conclusion

In the present study, we found that TRIM37 catalyzed the K63 chain and promoted the translocation of IKKγ by interacting with TRAF6, enhancing the activity and phase of the IKK complex in hepatic I/R injury. Our results might provide a potential target in hepatic I/R injury.

## Supplementary Information

**Additional file 1: Figure S1**. A-D, Suzuki score of liver after downregulating TRIM37 or TRAF6. E-F, transfection of si-TRAF2/4/6 in cells and tissues. G, transfection of adv-TRIM37 in cells and tissues. H, immunoflourence of p65.

## Data Availability

All origin data and materials are available.
