# Supplementary figures and images for: TRIM37 exacerbates hepatic ischemia/reperfusion injury by facilitating IKKγ translocation
Source: Mol Med. 2023 May 8;29:62. doi: 10.1186/s10020-023-00653-2 (PMC10165779; doi:10.1186/s10020-023-00653-2)

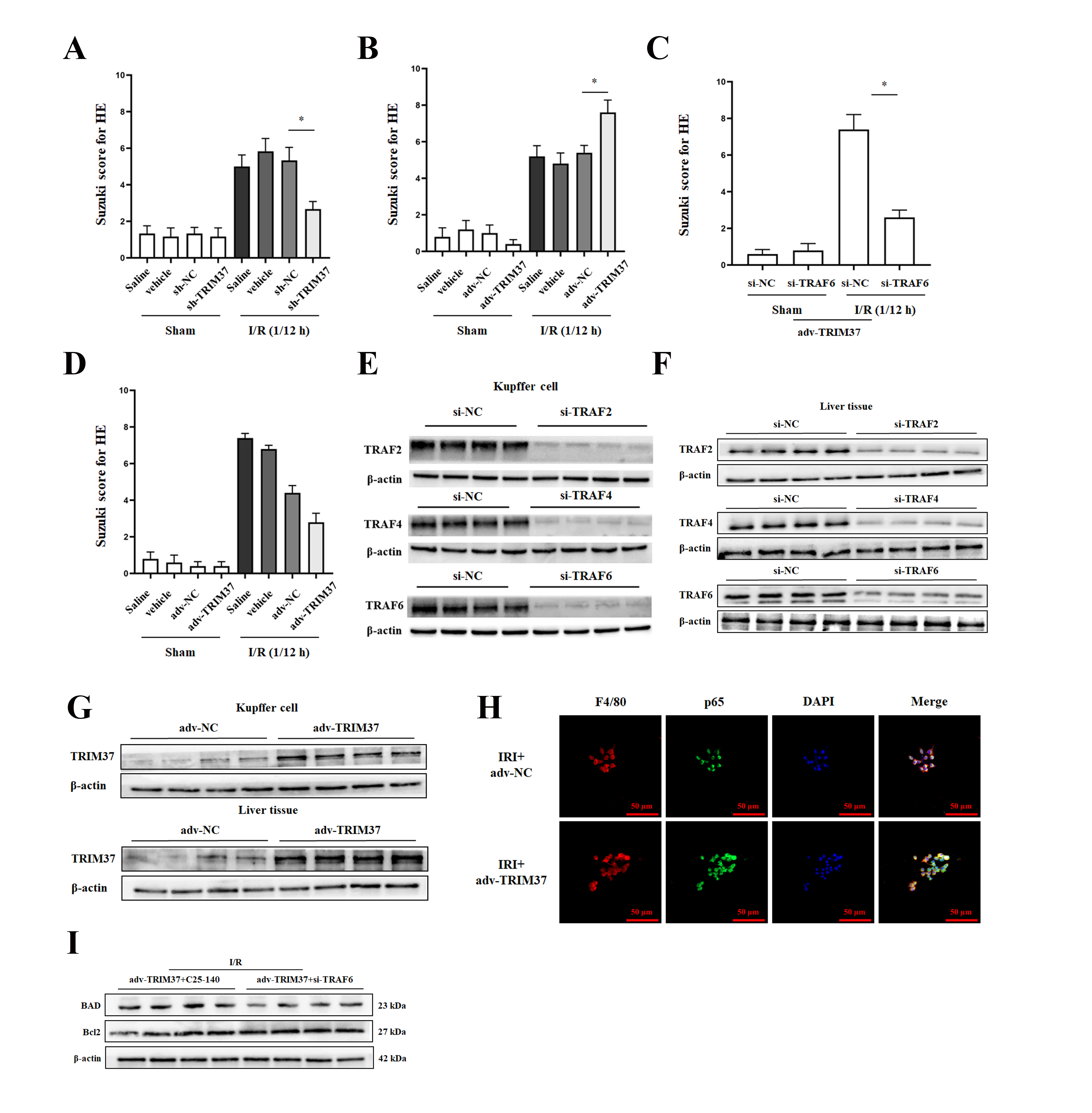

Supplement: Supplementary file 1 — Additional file 1: Figure S1. A-D, Suzuki score of liver after downregulating TRIM37 or TRAF6. E-F, transfection of si-TRAF2/4/6 in cells and tissues. G, transfection of adv-TRIM37 in cells and tissues. H, immunoflourence of p65. [file 10020_2023_653_MOESM1_ESM.tif]
